# Adeno-associated Virus (AAV) Capsid Chimeras with Enhanced Infectivity Reveal a Core Element in the AAV Genome Critical for both Cell Transduction and Capsid Assembly

**DOI:** 10.1128/JVI.02023-20

**Published:** 2021-03-10

**Authors:** Lydia Viney, Tilmann Bürckstümmer, Courtnee Eddington, Mario Mietzsch, Modassir Choudhry, Tom Henley, Mavis Agbandje-McKenna

**Affiliations:** a Intima Bioscience, New York, New York, USA; b Department of Biochemistry and Molecular Biology, Center for Structural Biology, McKnight Brain Institute, University of Florida, Gainesville, Florida, USA; Cornell University

**Keywords:** AAV, capsid, chimera, mutations, MOI, CRISPR, gene therapy, T cells, immunotherapy, adeno-associated virus

## Abstract

Adeno-associated viruses (AAVs) have attracted significant attention in the field of gene and cell therapy due to their use in highly effective delivery of therapeutic genes into human cells. The ability to generate recombinant AAV (rAAV) vectors comprising unique or substituted protein sequences has led to the development of capsid variants with improved therapeutic properties. Seeking novel AAV vectors capable of enhanced transduction for therapeutic applications, we have developed a series of unique capsid variants termed AAV *X-Vivo* (AAV-XV) derived from chimeras of AAV12 VP1/2 sequences and the VP3 sequence of AAV6. These AAV variants showed enhanced infection compared to the wild-type parental viruses of human primary T cells, hematopoietic stem cells (HSCs), and neuronal cell lines, and superiority over AAV6 for genomic integration of DNA sequences by AAV alone or in combination with CRISPR gene editing. AAV-XV variants demonstrate transduction efficiency equivalent to AAV6 but at 100-fold-lower multiplicities of infection (MOI), enabling T cell engineering at low AAV doses. The protein coding sequence of these novel AAV chimeras revealed disruptions within the assembly-activating protein (AAP), which likely accounted for the observed lower virus yield. A series of genome alterations, reverting the AAP sequence back to that of wild-type AAV6, had a negative impact on the enhanced transduction seen with AAV-VX, indicating overlapping functions within this sequence for both viral assembly and effective T cell transduction. Our findings show these AAV-XV variants are highly efficient at cell transduction at low doses and demonstrate the importance of the AAP coding region in both viral particle assembly and cell infection.

**IMPORTANCE** A major hurdle to the therapeutic potential of AAV in gene therapy lies in achieving clinically meaningful AAV doses, and secondarily, the ability to manufacture commercially viable titers of AAV to support this. By virtue of neutralizing antibodies against AAV that impede patient repeat dosing, the dose of AAV for *in vivo* gene delivery has been high, which has resulted in unfortunate recent safety concerns and deaths in patients given higher-dose AAV gene therapy. We have generated new AAV variants possessing unique combinations of capsid proteins for gene and cell therapy applications termed AAV-XV, which have high levels of cell transduction and gene delivery at lower MOI. Furthermore, we demonstrate a novel finding, and an important consideration for recombinant AAV design, that a region of the AAV genome encoding the capsid viral protein and AAP is critical for both virus yield and the enhancement of infection/transduction.

## Abstract (precis)

A major hurdle to the therapeutic potential of AAV in gene therapy lies in achieving clinically meaningful AAV doses, and secondarily, the ability to manufacture commercially viable titers of AAV to support this. By virtue of neutralizing antibodies against AAV that impede patient repeat dosing, the dose of AAV for *in vivo* gene delivery has been high, which has resulted in unfortunate recent safety concerns and deaths in patients given higher-dose AAV gene therapy.

## INTRODUCTION

The adeno-associated viruses (AAVs) are among the most widely developed and actively studied vehicles for gene and cell therapy and have shown remarkable promise in numerous clinical trials for multiple human disorders (1). As vectors for gene delivery into human cells, these single-stranded DNA viruses show broad tropism, with multiple serotypes identified for transducing cells from many different tissue types (1). Their nonpathogenicity, coupled with long-term transgene expression, make AAVs attractive as a therapeutic technology for *in vivo* gene therapy (2). Despite broadly low innate immunogenicity, concerns over humoral immune responses against AAV capsids, observed in recent clinical trials, have been raised and are particularly associated with high vector doses (3, 4). This *in vivo* limitation, as well as the high doses or multiplicities of infection (MOI) of virus required for sufficient cell transduction and the need to expand the repertoire of transducible tissue types addressable with AAV, is motivating further development of recombinant AAV (rAAV) technology.

There are 13 naturally occurring AAV serotypes and numerous AAV isolates (5), each with unique capsid viral protein (VP) sequences and transduction profiles in different tissues (6). As an example, AAV6 is consistently better than other serotypes in *ex vivo* transduction of human immune cells (7, 8). Each serotype’s distinct VP sequences assemble in a strict T=1 icosahedral arrangement that enables packaging of the AAV genome into an infectious virion (9). Novel variants of AAV are also being identified from sequencing experiments in different cell types, such as within CD34^+^ hematopoietic stem cells (HSCs) (10). Furthermore, uniquely engineered AAV vectors with enhanced transduction efficiencies have been developed (11) through capsid mutation by rational design (12, 13), by directed evolution (14), or by combining different serotypes through capsid shuffling (15). Thus, combining sequences from divergent serotypes, or specific mutations of surface-exposed capsid residues known to facilitate viral entry into cells, may be an effective route to improve the infectious properties of AAV.

While AAV vector transduction can lead to high levels of transient transgene expression by episomal genomes, integration into the host genome typically occurs at a very low frequency (16). The stable genomic integration of AAV donor vectors can be increased significantly via the combination of AAV vectors with CRISPR/Cas9 gene editing (17). A targeted double-strand break (DSB) introduced by Cas9 at a specific location within the genome can be effectively repaired with an AAV template designed with homology to the target locus, via the pathway of homology-directed repair (HDR) (17). This AAV plus CRISPR combination approach has been effectively used by us and by others to perform genetic engineering of difficult-to-target cell types such as primary human T cells at levels of efficiency that are therapeutically relevant (18). However, despite the advances in AAV vector engineering, capsid evolution, and use of synergistic technologies such as CRISPR, high-AAV-dose MOI, typically 1 × 10^6^ virus particles per cell, are still required for gene delivery into cells being modified for either research or therapeutic applications (19). This makes AAV a costly technology to deploy at scale for cell therapies and, as mentioned, may preclude *in vivo* efficacy due to the potentially toxic high doses required for gene therapy.

To address the above limitations, novel AAV capsid variants (AAV-XV) with enhanced transduction of human T cells were developed to improve the efficiency of *ex vivo* gene delivery. A series of capsid variants were engineered, via rational design, by substituting the VP1 unique (VP1u) and VP1/2-common region sequences of AAV6 with those from divergent AAV serotypes AAV4, AAV5, AAV11, and AAV12 to create chimeric AAV6 vectors. Analysis of the resulting chimeras, for performance in transduction assays using primary human T cells found several variants that achieved levels of transduction 100-fold higher than those of wild-type AAV6 at a similar MOI or 10- to 30-fold higher at 2-log lower doses. This enhanced transduction was further seen in other tissue types, including hematopoietic stem cells and neuronal cells.

Analysis of the sequences of the AAV-XV chimeras identified an overlapping region of the *cap* open reading frame (ORF) encoding VP2 and the assembly-activating protein (AAP) as being critical for both optimal vector yield and efficient cellular infectivity. Our data demonstrate that the AAV-XV variants are highly efficient at cellular transduction of different cells and broadly relevant for the *ex vivo* engineering of human T cells for gene and cell therapy and have potential as efficient AAV vectors for *in vivo* gene delivery at low doses.

## RESULTS

### Generation of new chimeric AAV6 capsid variants.

**FIG 1:**
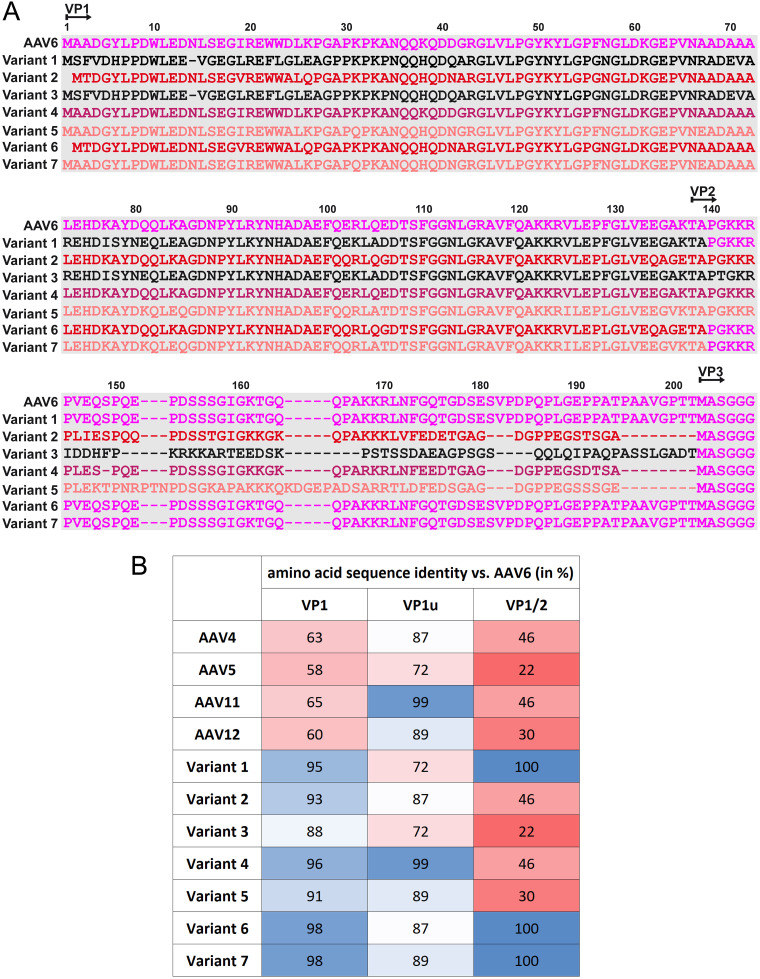
Amino acid capsid sequence comparison. (A) Amino acid sequence alignment of wild-type AAV6 and the chimera variants. The VP1, VP2, and VP3 N termini and the residue numbers based on AAV6 VP1 are indicated above the amino acid sequence. (B) The amino acid sequence identities of AAV4, AAV5, AAV11, AAV12 and the variants to the AAV6 VP1, VP1u, and VP1/2 common regions, respectively, are given as percentages.

**FIG 2:**
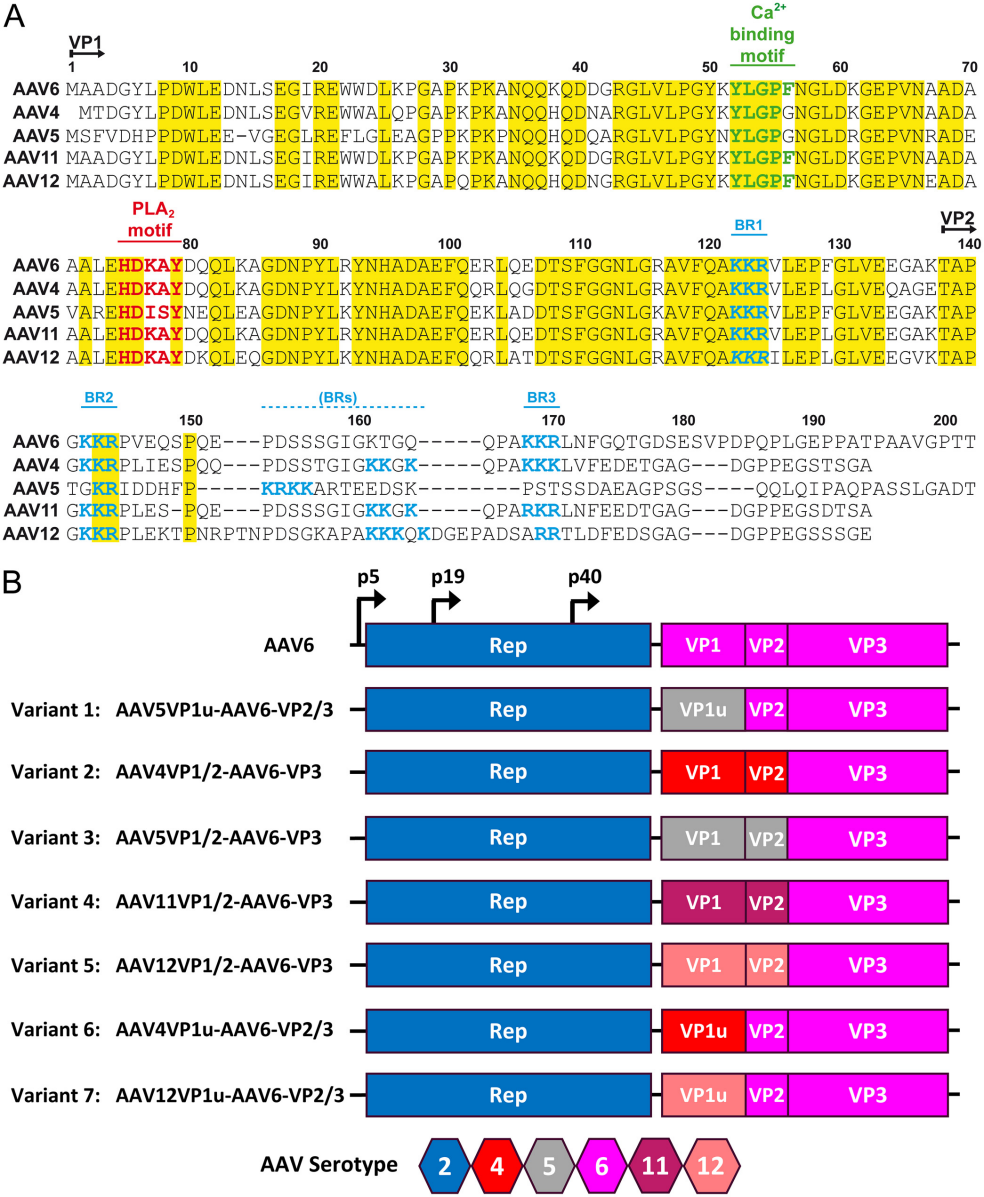
Design of recombinant AAV6 capsid variants. (A) Alignment of the VP1u and VP1/2 common region sequences of AAV4, AAV5, AAV6, AAV11, and AAV12 showing conservation of functionally important motifs. (B) Schematic diagram of the AAV2 Rep and the AAV serotypes contributing VP1, VP2, and VP3 to the AAV6 variants. The approximate positions of promoters are indicated. A serotype color key is provided.

To engineer capsid variants with the potential to transduce human cells at high efficiencies, 7 chimeric AAV capsid sequences, for which AAV6 provided the VP3, were generated (Fig. 1A). Each variant incorporated sequence from the VP1u or VP1u+VP1/2 common regions of AAV4, AAV5, or AAV12 or the VP1u+VP1/2 common region of AAV11. The serotypes substituted represent the most diverse based on the pairwise comparison of the VP1u and VP1/2 common regions from AAV1 through AAV13 with AAV6 (Fig. 1B), with the intent to create maximum diversity in the resulting chimeras. A multiple sequence alignment between the selected serotypes and AAV6 showed conservation of functional regions that included a PLA2 motif (20), a calcium binding motif (21), and basic residue clusters that serve as nuclear localization sequences (NLS) (22), although positioning of the latter was different for AAV5 (Fig. 2A). This alignment showed a higher sequence identity for the VP1u region than for the VP1/2 common region (Fig. 1B). Recombinant AAV vectors utilizing wild-type AAV6 and the 7 variant capsids (Fig. 2B), packaging a donor template with a nanoluciferase (NanoLuc) luciferase gene, were produced and evaluated for their performances as *ex vivo* gene delivery vectors in human T and stem cells and *in vitro* in neuronal cells.

### Chimeric AAV6 capsid variants outperform the wild-type as donors for CRISPR genomic integration in human T and stem cells at a low MOI.

**FIG 3:**
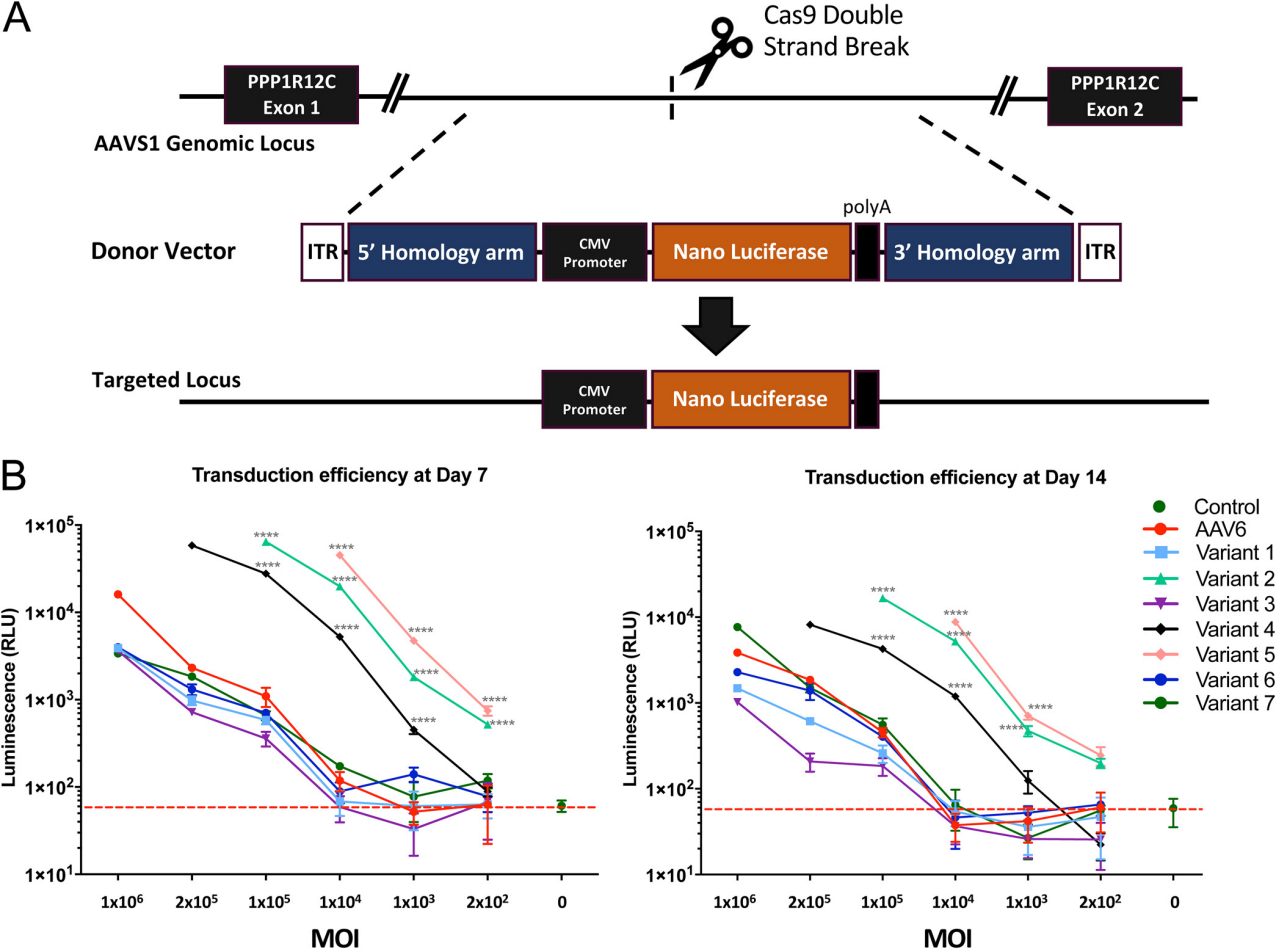
Transduction efficiency and CRISPR-mediated genomic integration of AAV6 capsid variants in human T cells. (A) Schematic of the AAV6 targeting vector for the integration of a luciferase expression cassette into the PPP1R12C (AAVS1) genomic locus. Diagram indicates the relative position of CRISPR-mediated genomic cleavage and the resulting modified locus upon homology-directed repair with the AAV6 luciferase vector. (B) Multiplicity of infection (MOI) dose titration of wild-type AAV6 and chimeric AAV6 capsid variants measuring CRISPR-mediated genomic integration of a luciferase reporter gene in human CD3^+^ T cells at 7 and 14 days post-infection. Statistical significance was determined by a one-way analysis of variance (ANOVA) test for multiple comparisons. ****, *P* < 0.0001.

To assess the impact of the VP sequence substitutions into AAV6 on infectivity and genomic integration efficiencies, CD3^+^ T cells were electroporated with Cas9 mRNA and a single guide RNA (sgRNA) targeting the AAVS1 locus, followed by infection with the rAAV chimeras. The rAAV vectors delivered a NanoLuc expression cassette flanked by homology arms, allowing targeted integration at the AAVS1 locus (23) (Fig. 3A). An MOI titration of these variants monitored at 7 and 14 days post-infection and CRISPR transfection showed 3 of the 7 chimeras (variants 2, 4, and 5), with significant enhancement of transduction and genomic integration of the targeting construct (1.5 to 2 log) compared to those of wild-type AAV6 (Fig. 3B). Interestingly, at a low MOI of 1 × 10^4^ genome copies per cell (gc/cell), the best variant, variant 5, achieved luciferase levels that required a 100-fold higher MOI of wild-type AAV6 vectors (1 × 10^6^). Variants 2, 4, and 5 possess the VP1u+VP1/2 common regions of AAV4, AAV11, and AAV12, respectively (Fig. 1A and 2B) and, unlike AAV5 and AAV6, contain an additional basic cluster (Fig. 2A). In contrast, the variants containing only the VP1u region of AAV4, AAV11, and AAV12, respectively, did not display enhanced transduction and genomic integration, suggesting that their VP1/2 common region is the determinant of this phenotype (Fig. 2B and 3B). Notably, the variant with a substituted VP1+VP1/2 common region of AAV5 (variant 3) did not enhance transduction and was arguably the worst-performing vector (Fig. 3B).

**FIG 4:**
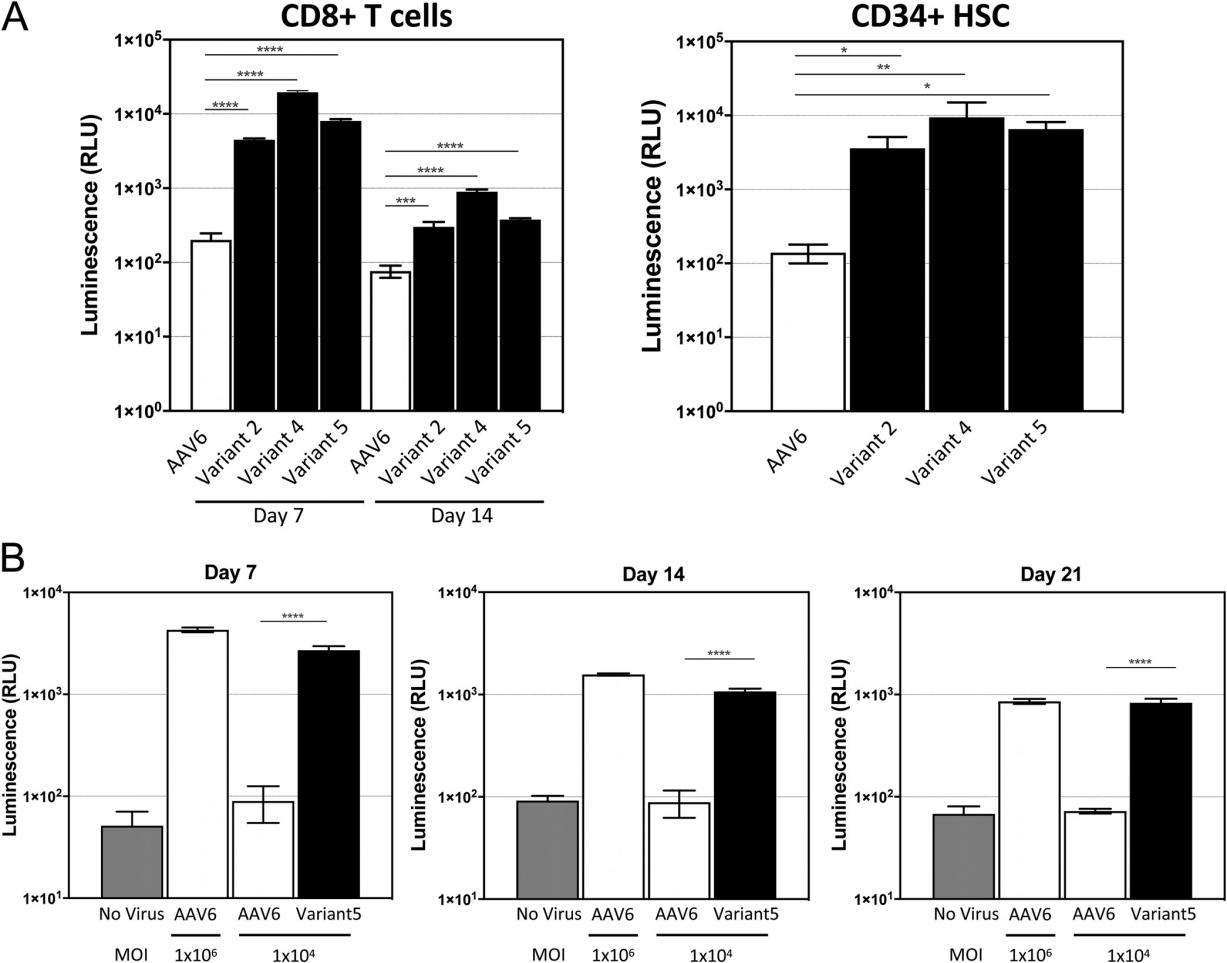
Low-MOI transduction of human CD8^+^ T cells and hematopoietic stem cells (HSCs) by AAV6 capsid variants. (A) Comparison of CRISPR-mediated genomic integration by wild-type AAV6 and the three top-performing AAV6 capsid variants at a low MOI (1 × 10^4^/cell) in primary CD8 T cells (left) and human CD34^+^ HSCs (right). (B) Comparison of transduction efficiency of capsid variant 5 (AAV12VP1/2-AAV6VP3) at a low MOI (1 × 10^4^/cell) and wild-type AAV6 at a high MOI (1 × 10^6^/cell) in CD8 T cells over a 3-week period in the absence of CRISPR gene editing. Statistical significance was determined by an unpaired *t* test or by one-way ANOVA test for multiple comparisons. *, *P* < 0.05; **, *P* < 0.01; ***, *P* < 0.001; ****, *P* < 0.0001.

Further analysis of the 3 best-performing capsid variants in both human cytotoxic CD8^+^ T cells and CD34^+^ HSCs confirmed higher transduction efficiencies and luciferase targeting to the AAVS1 genomic site of up to 2 log (Fig. 4A). This enhanced performance was also observed in the absence of a CRISPR-mediated DSB, with variant 5 showing a 1-log increase in transduction efficiency over AAV6 when measured up to 21 days post-infection (Fig. 4B). Again, this variant achieved similar levels of T cell transduction at the lower MOI of 1 × 10^4^ gc/cell as AAV6 at an MOI of 1 × 10^6^ gc/cell. This AAV-XV variant thus demonstrated superior infectivity and transduction performance at low doses to those of the wild-type AAV6 sequence from which it was derived.

### Transduction efficiency was inversely correlated with variant yield.

**FIG 5:**
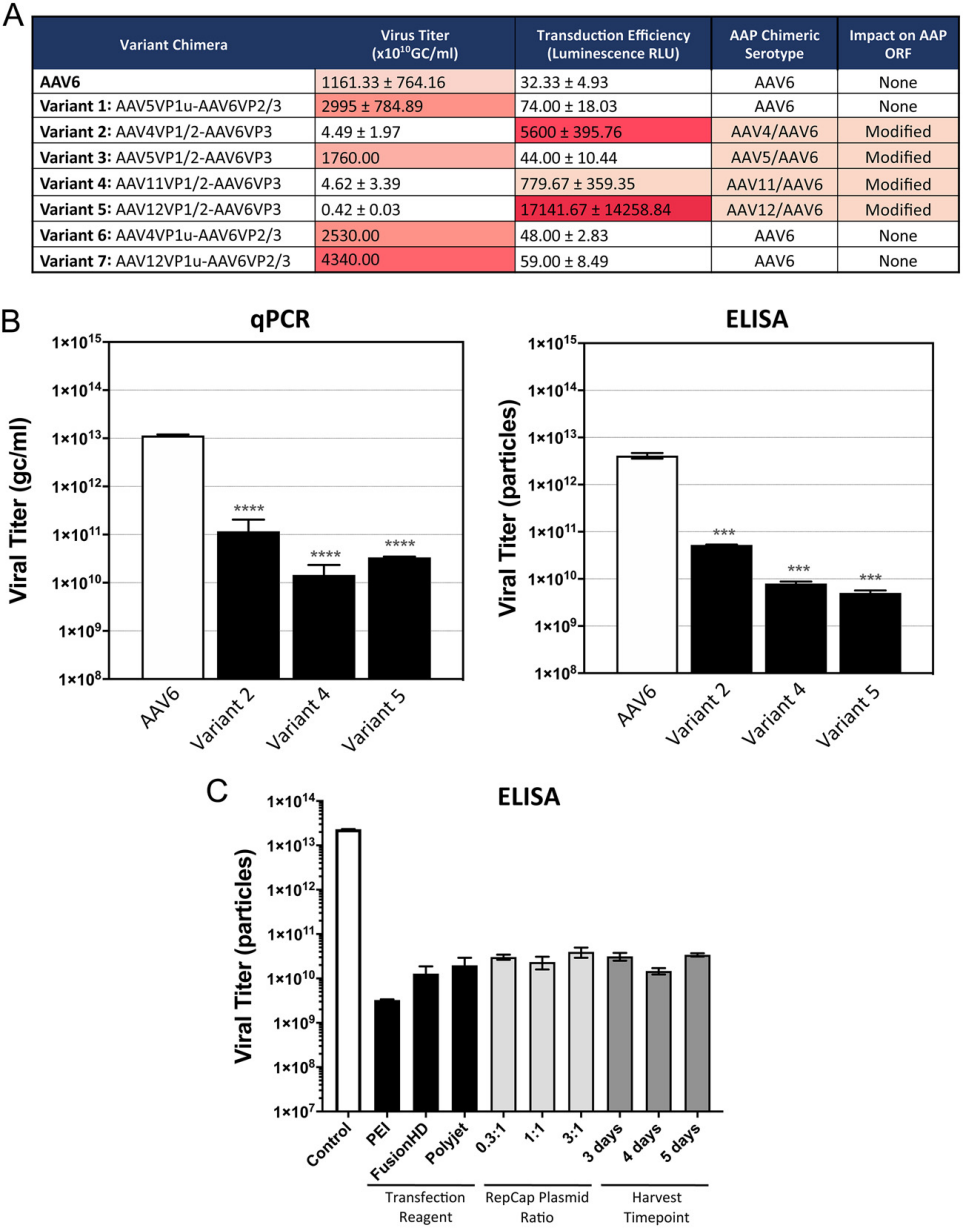
AAV6 capsid variants are defective for capsid assembly. (A) Viral packaging titers and T cell-luciferase transduction values for AAV6 capsid variants and the impact of the chimeric capsid sequences on the coding protein sequence of AAP. A high rate of transduction correlates with a low titer. Heat map coloring is used to indicate the magnitude of yield and transduction. Viral preparations for AAV6 and variants 2, 4, and 5 were produced 3 times, variant 1 was produced 2 times, and variants 3, 6, and 7 were produced once. (B) Viral packaging titers from purified AAV particle yield measured by quantitative PCR (qPCR) and enzyme-limited immunosorbent assay (ELISA). Both are reduced for variants 2, 4, and 5 compared to wild-type AAV6. (C) Modifying transfection conditions had no effect on AAV6 variant capsid yield. Changing the transfection reagent, the ratio of AAV helper plasmid transfected, or the virus harvest time posttransfection did not lead to any increase in viral genome or particle titer (as measured above) for variant 5. Wild-type AAV6 and variant 5 genome and particle titers were measured from purified particles. Statistical significance was determined by an unpaired *t* test or by one-way ANOVA test for multiple comparisons of results in triplicate or duplicate replicas. ***, *P* < 0.001; ****, *P* < 0.0001. GC, genome copies; RLU, relative light units.

Several of the capsid chimeras routinely produced viral titers lower than those of wild-type AAV6 from packaging cell lines, while other variants showed no detectible reduction (Fig. 5A). When the T cell transduction performance of each capsid variant was compared to its viral titer, those of the 3 best-performing variants, 2, 4, and 5, were on average 3 log lower than that of wild-type AAV6. Comparison of the viral titer of the purified wild-type AAV6 and these three capsid variants by quantitative PCR (qPCR) after DNase I treatment showed significantly lower titers, by 2 to 3 log, for the three variants (Fig. 5B). Quantification of viral capsids by enzyme-limited immunosorbent assay (ELISA), using an antibody recognizing an epitope present on AAV6 and all capsid variants, confirmed that all variants showed a reduced number of viral particles, also at 2 to 3 log less than the number for wild-type AAV6 (Fig. 5B). This observation indicated that the capsid substitutions in variants 2, 4, and 5 impacted either capsid assembly or the stability of assembled viral particles.

Attempts to improve variant 5 capsid yield by optimizing the AAV vector transfection method, changing the ratio of AAV helper and AAV donor plasmid transfected, or by extending duration of cell transfection prior to harvesting from the supernatant failed to demonstrate any detectible increase in viral particle titer (Fig. 5C). All conditions tested resulted in similar vector yield as measured by qPCR (not shown) and similar average purified particle yield as measured by ELISA (Fig. 5C). Capsid yield for variant 5 remained ∼2 to 3 log lower than that for wild-type AAV6. To understand the mechanistic reason for this impairment in particle assembly, the region of the AAV6 capsid sequence altered in these capsid chimera variants was investigated.

### The AAP sequence has an impact on the transduction efficiency of AAV6 capsid chimeras.

**FIG 6:**
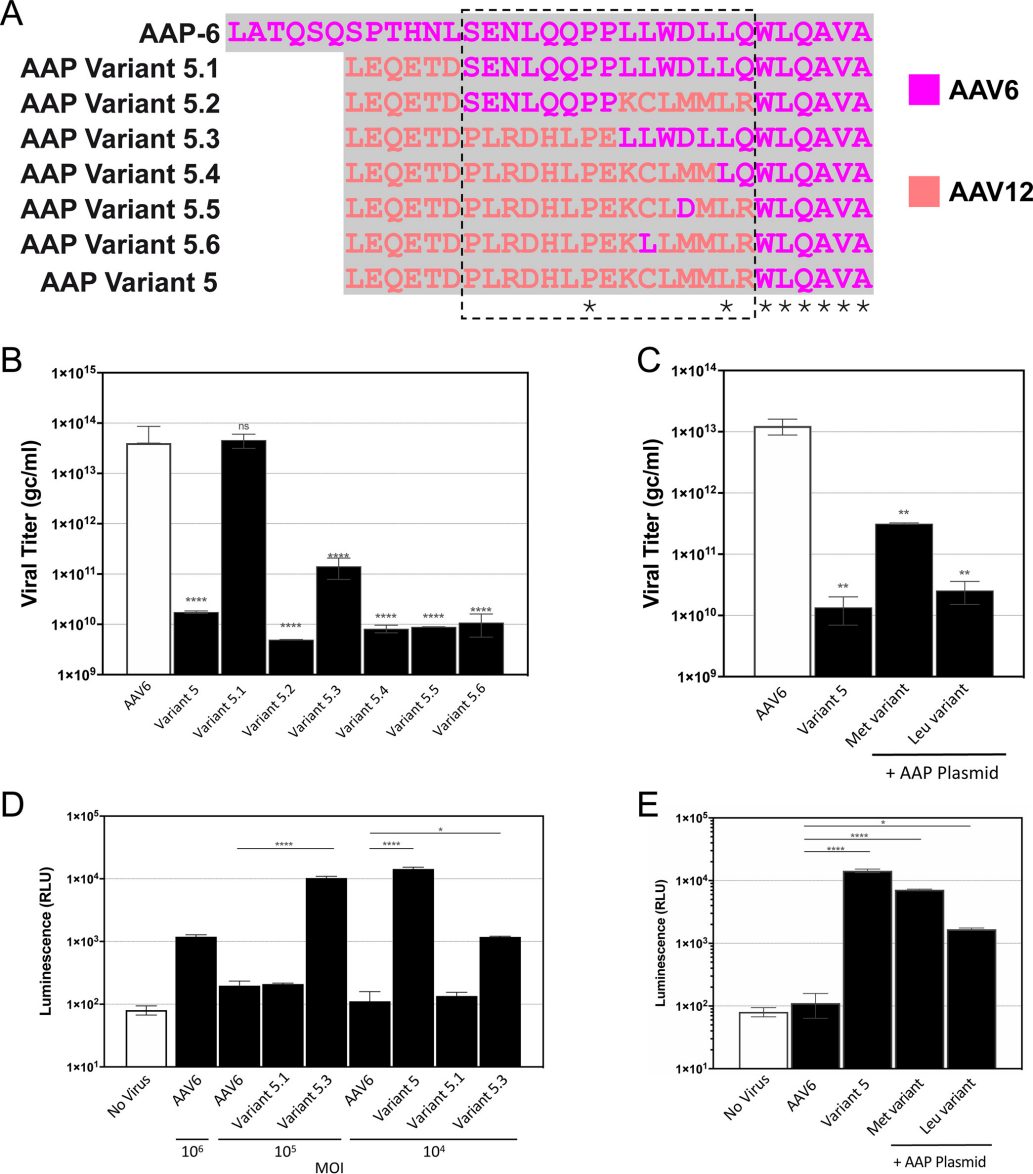
The AAP sequence plays a role in gene expression in addition to capsid assembly. (A) Amino acid sequence alignment of the AAP sequences at the VP2/VP3 boundary for wild-type AAV6 and capsid variant 5 modifications. The wild-type AAV6 sequence is at the top; capsid variant 5 AAPs with changes reverting to the wild-type sequence at amino acids 13 to 27 (outlined by the dashed-line box) of AAP are labeled 5.1 to 5.6. (B) Genome titers for wild-type AAV6, capsid variant 5, and variants 5.1 to 5.6 with altered AAP sequences. (C) Genome titers of wild-type AAV6 and of capsid variant 5 alone and with cotransfection of two forms of the full-length AAP construct in the packaging cells, one starting with a methionine and the other a leucine. (D) AAP sequence alterations play a role on transduction of primary CD8^+^ T cells. Viruses are as in panel C. Complete correction of amino acids 13 to 27, as in variant 5.1, decreases T cell transduction levels, whereas partially corrected 5.3 along with the original variant 5 surpasses wild-type AAV6 at a low MOI. (E) T cell transduction by variant 5 packaged by cotransfection of the AAP expression constructs (as in panel D). Statistical significance was determined by an unpaired *t* test or by one-way analysis of variance (ANOVA) for multiple comparisons of results in triplicate or duplicate replicas. *, *P* < 0.05; **, *P* < 0.01; ***, *P* < 0.001; ****, *P* < 0.0001.

Given the importance of AAP for AAV capsid assembly (24, 25), the alterations in the sequences of each of the capsid variants within their cap ORF coding for AAP was analyzed. In addition to the VP sequence, the AAP sequence was changed relative to that of wild-type AAV6 in 4 of the 7 capsid chimeras containing the VP1u+VP1/2 common region (Fig. 6A and data not shown), including in the 3 variants showing enhanced transduction at low MOI (Fig. 5A). Previous studies identified the importance of the AAP N terminus in capsid stability and assembly (26, 27). Thus, to restore a potentially lost AAP function for variant 5, a series of substitution mutants was generated in which the AAP-12 residues equivalent to AAP-6 positions amino acids (aa) 13 to 27 were gradually reverted back to AAV6 (Fig. 6A). A complete rescue of viral titer to the levels of wild-type AAV6 was achieved by reintroducing AAP-6 (variant 5.1) (Fig. 6B). This observation confirmed the hypothesis that variant 5 has an assembly defect due to compromised AAP function. An approximately 1-log rescue was observed in vector production, compared to variant 5, when AAP-12 sequences corresponding to AAP-6 residues aa 21 to 27, the start of the AAP-6 hydrophobic region, were reintroduced to the chimera (variant 5.3). The remaining substitution variants could not effectively rescue the assembly defect (Fig. 6B).

We further demonstrated that cotransfection with a functional full-length cytomegalovirus (CMV) promoter-driven AAP-6 gene into the producer cells, in *trans*, along with variant 5 Rep/Cap and donor vector, rescued titer by up to 1.5 log (Fig. 6C). The AAP gene with the natural CTG start codon (leucine) present in the AAP-6 sequence was insufficient to provide this rescue, while substitution to an ATG start codon to code a methionine produced the titer rescue (Fig. 6C). This likely reflects the requirement to have a canonical start signal for robust translation initiation and the expression of AAP when present within an expression plasmid in HEK293 packaging cells.

Finally, the AAV12VP1/2-AAV6VP3 variant that was fully (variant 5.1) or partially (variant 5.3) rescued by AAP-6 sequence restoration were assessed for T cell transduction and genomic integration of the luciferase gene when codelivered with CRISPR (Fig. 6D). Variant 5.1, in which AAP-6 residues aa 13 to 27 had been reverted to AAV6, gave the same level of transduction as AAV6 at all MOI compared, and it had thus lost any enhancement in infectivity or transduction (Fig. 6D). In contrast, variant 5.3, in which only aa 21 to 27 of AAP-6 were reintroduced, maintained some level of superiority over AAV6, resulting in a 2-log higher transduction at an MOI of 1 × 10^5^ gc/cell and a 1-log higher transduction at the lowest MOI tested of 1 × 10^4^ gc/cell. Variant 5 maintained superiority to AAV6 at an MOI 1 × 10^4^ gc/ml (Fig. 6D).

Variant 5 vectors, generated with the Met-AAP-6 cotransfection construct, resulted in levels of T cell transduction approximately 2-log higher than that of AAV6, and almost as high as the level observed for variant 5 that is AAP disrupted (Fig. 6E). The variant 5 sample produced by Leu-AAP-6 cotransfection shows transduction that is ∼1 log higher than that of wild-type AAV6 (Fig. 6E). Collectively, these data demonstrate that a modified AAP sequence in the capsid chimera variants can restore vector production at the expense of transduction efficiency at lower MOI. However, cotransfection with AAP in *trans* during vector production also partially restores packaging (>1 log), with minimal impact on transduction at a low MOI (Fig. 6C and E).

### AAP is required for the production of the chimeric capsid variants.

**FIG 7:**
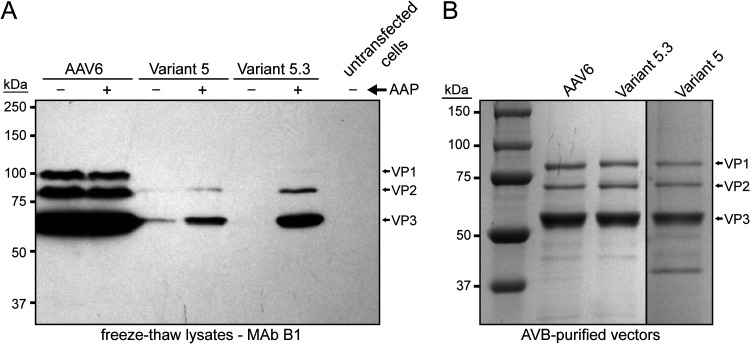
AAP is required for production of AAV-XV capsid variants. (A) Western blot analysis of freeze-thawed lysates from transfected cells with the AAV-XV variant constructs in presence or absence of AAP. The AAV VPs as labeled on the right were detected with monoclonal antibody (MAb) B1. (B) SDS-PAGE of AVB-purified AAV6, variant 5, or variant 5.3 vector preparations.

The expression of VP1, VP2, and VP3 in the producer cells was analyzed by Western blotting for AAV6 and variants 5 and 5.3 from preps generated with or without cotransfection of the AAP expression construct. As expected, abundant levels of the three AAV6 capsid proteins could be readily detected in the cell lysates, whereas very low to undetectable levels of VP1 to VP3 were observed with both variants 5 and 5.3 in the absence of cotransfected AAP (Fig. 7A). When AAP was provided in *trans*, increased levels of VP expression could be detected in variant 5 preps and even higher levels in variant 5.3 preps, and in each case VP1 expression was too low to visualize. However, when the capsid proteins were analyzed from purified vectors, all three VP proteins were detectable at the expected ratio of approximately 1:1:10 for wild-type AAV6 and variants 5 and 5.3 (Fig. 7B). Taken together, these data demonstrate that capsid proteins are expressed at lower abundances in the producer cell for the AAV-XV variants and that AAP is required for successful production of the chimera variants.

### Enhanced transduction of neuronal cell types by chimeric AAV6 capsid variant 5.3.

**FIG 8:**
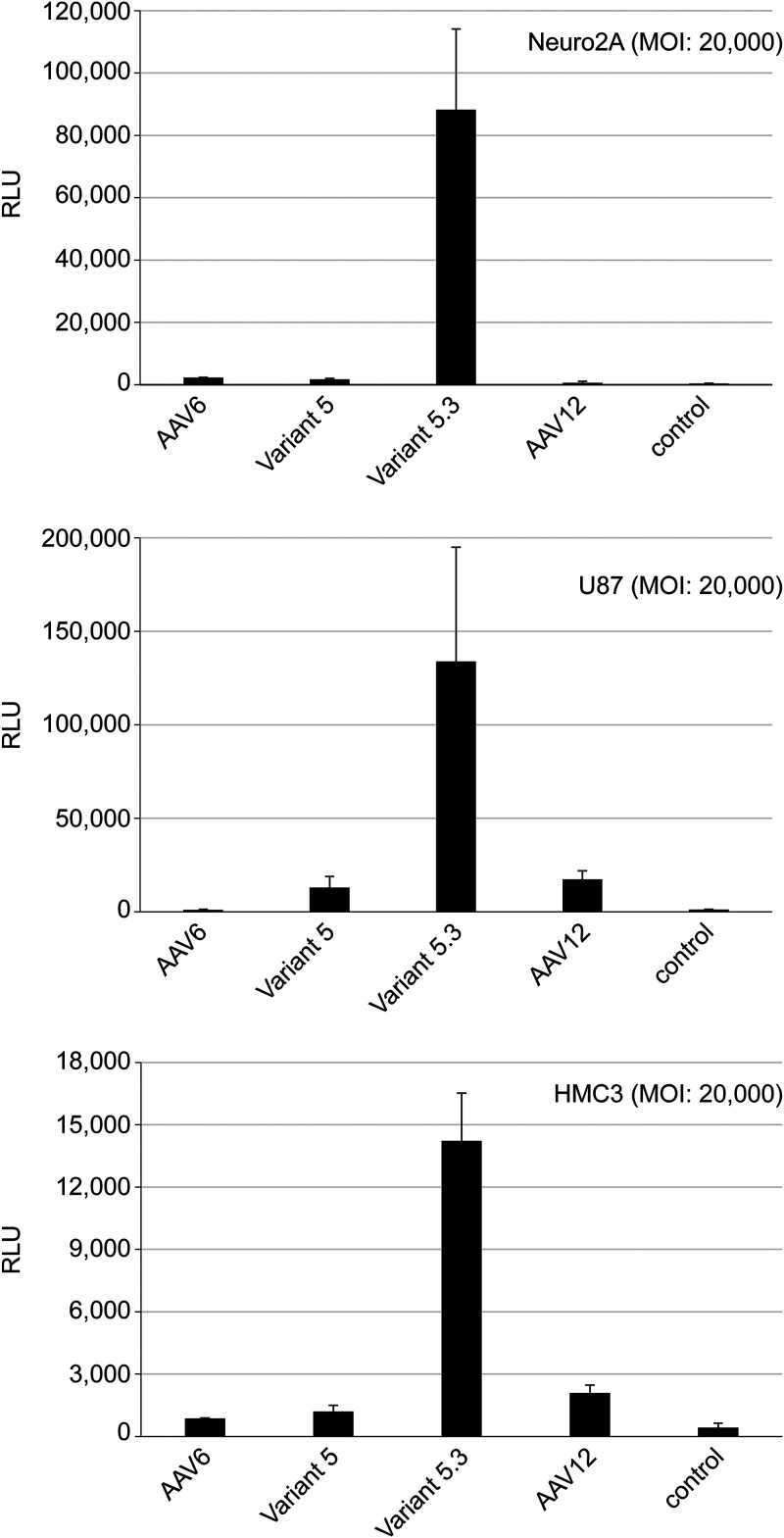
Efficient transduction of neuronal cell lines by AAV6 capsid variant. Analysis of Neuro2A, U87, and HMC3 cell transduction by AAV6, AAV12, variant 5, and variant 5.3 luciferase vectors at an MOI of 2 × 10^4^ at 2 days post-infection. The transduction efficiency was determined by a luciferase assay. RLU, relative light units.

Given the enhanced transduction of T cells and HSCs seen at a low MOI with capsid variants 5 and 5.3, we extended the analysis to other cell types to investigate their transduction efficiency. Three cell lines, Neuro2A, U87, and HMC3, derived from central nervous system (CNS) tissue infected with AAV6, AAV12, and the chimera variants at an MOI of 2 × 10^4^ gc/cell, revealed an enhanced transduction by variant 5.3 (Fig. 8). With an MOI of 2 × 10^4^, the transduction of these cells with AAV6, AAV12 or variant 5 remained low, suggesting a unique attribute of the variant 5.3 capsid that potentiates the transduction of cells of neuronal origin.

## DISCUSSION

Recombinant AAV vectors hold great promise as a gene delivery vehicle (1, 2). Despite demonstrated clinical efficacy reported for several indications, limitations remain that impede the broader applicability of the AAV technology for efficient and persistent gene delivery to many cell types. The low-frequency genomic integration of AAV had previously made the genetic engineering of cells a difficult and laborious task that required the use of drug selection genes or fluorescent markers to select the small number of cells successfully modified with the AAV donor (28). The advent of designer nuclease technologies and the combination of CRISPR gene editing with AAV enabled substantially higher levels of targeted genome integration of AAV donors and opened up the ability to genetically modify cell types such as primary human T cells for therapeutic applications (17, 18). However, even with these advances in technology, high-MOI doses of AAV are still required to reach sufficient levels of transgene integration. Despite optimized production procedures, the high dose requirements of AAV are expensive and limit scaling to clinical manufacture. Perhaps even more importantly, recent evidence of immune responses directed toward large quantities of AAV administered *in vivo* to patients in gene therapies highlights the potential toxicity issues associated with high AAV doses (3, 4). In an effort to expand and improve the transduction efficiency and genetic engineering potential of AAV, our chimeric AAV variant design approach combining VPs from several divergent serotypes has generated new AAV chimeras (AAV-XV variants) with enhanced transduction characteristics for human cells that address the requirement for a high MOI. AAV6 is considered the best serotype for the transduction of T cells and thus served as a starting point to evaluate the impact of VP1 and VP2 swapping (Fig. 1 and 2) in an attempt to identify modifications that further improved this T cell tropism (19).

Collectively, our data show that the engineered capsid chimeras, comprising AAV12VP1/2-AAV6VP3, can transduce T cells at 2-log lower MOI doses than those for wild-type AAV6 with equal efficiency. Superior transduction and stable gene delivery were observed for AAV-XV variants compared to AAV6 (Fig. 3 and 4), when used as a donor template in combination with CRISPR gene editing or as a method for AAV-mediated homologous recombination in the absence of targeted genomic cleavage. In addition to human T cells, we find that these novel variants show improved transduction in human stem cells and various neuronal cells derived from different neurological tissues, demonstrating potential broad effectiveness and tropism in multiple tissue types important for gene therapy (Fig. 4A and Fig. 8).

While the mechanism for this enhanced transduction is not yet clear, the enhanced transduction efficiency requires the C-terminal amino acid sequences of the AAV12 VP1/2 common region. The VP1/2 common region and the N terminus of VP3 are believed to be located in the interior of the capsid and to become externalized to the capsid surface upon acidification of the endosome during cellular trafficking (29). Thus, the observed enhanced efficiency of infection is likely to be a postentry effect due to improved interaction with trafficking receptors/effectors. Furthermore, this region has been described as structurally highly flexible (30) and no significant structural differences are predicted (http://original.disprot.org/) by substituting AAV6 to AAV12 sequences.

What is clear from the data is that the modification of the AAV6 capsid sequence to incorporate the VP1/2 of AAV12 results in a change of the amino acid sequence for the overlapping AAP. This is likely the reason for the lower titer yield for the AAV vectors with the variants compared to that of the wild-type AAV6, given the well-characterized assembly-promoting activity of AAP and the identification of mutations within this sequence that reduce the interaction of AAP with the capsid, impairing its ability to promote particle assembly (24–26, 31, 32). The critical region affected in the AAV-XV variants lies within a hydrophobic region of AAP, and aa 13 to 27 appear particularly important for stability and assembly functions (26). The data demonstrate that the defect can also be partially rescued by restoring aa 21 to 27 to AAP-6, supporting the theory that these capsid changes impaired AAP-mediated capsid assembly.

While the restoration in titer via these amino acid reversions is significant, analysis of capsid protein expression demonstrated impaired production that was robustly improved through coexpression of a functional AAP gene during particle assembly (Fig. 6B and Fig. 7). Intriguingly, while this partial AAP-rescued chimera (variant 5.3) retained significantly enhanced T cell transduction over that of AAV6, when tested in neuronal cell types at a low MOI, this variant outperformed the other tested variant and wild-type serotypes, indicating a potentially unique property of this capsid for transduction of cells derived from neuronal tissue (Fig. 8).

The overlapping ORF encoding the VPs and AAP adds complexity to the rational design of capsid variants (24). This was evident in the chimeras generated in this study, in which VP changes resulted in AAP modification and low vector yield. However, the ability to transduce cultured human cells of different tissue types at several-log-order-lower MOI to achieve efficient genomic integration of AAV donor DNA requires far less virus to be manufactured, achieving a balance between potency and yield. In conclusion, our data have shown that generation of AAV capsid chimeras by VP protein combinations from divergent serotypes is an effective approach for generating novel AAV variants with unique and enhanced functional properties for cellular transduction on multiple tissue types relevant for gene therapy. Careful consideration of the precise sequence changes is important given the overlapping nature of AAV ORFs, and particular care is needed to avoid detrimental modifications to the AAP or MAAP protein (33). The AAV-XV capsid chimeras, AAP rescued or not, shows the useful property of highly efficient transduction of cultured human cells at >100-fold lower MOI compared to that of the parental AAV6 and AAV12 from which they are derived. AAV-XV variants have novel properties for enabling low-dose gene delivery, thus enhancing the safety profile of AAV vectors administered *in vivo* to mitigate the toxicity that can occur with current AAV-based gene therapies at high doses.

## MATERIALS AND METHODS

### AAV variant design and plasmid generation.

AAV variants were designed by first extracting the sequences of the VP1u and VP1/2 common region from AAV1 through AAV13 (including AAVrh.10) and performing a pairwise alignment of each serotype to AAV6 using Clustal Omega (https://www.ebi.ac.uk/Tools/msa/clustalo/). The pairwise identity and similarity for each serotype sequence were compared, and serotypes with the lowest identities within the VP1u and VP1/2 common region (serotypes AAV4, AAV5, AAV11, and AAV12) were used to generate the chimeric sequences with the AAV6 VP3. At this point, these chimeras were called variants 1 to 7. DNA encoding the chimera regions was generated by DNA synthesis (GenScript Biotech) and subcloned into the AAV6 RepCap plasmid to replace the AAV6 sequence (Plasmid Factory).

A gene targeting vector to measure genome integration via AAV homologous recombination was constructed by flanking the NanoLuc luciferase gene under the control of the CMV promoter with 1-kb sequences homologous to a transcriptionally active intronic region within the human AAVS1 locus. This AAVS1 luciferase donor contained AAV2 inverted terminal repeats (ITRs) for packaging of the single-stranded vector into chimeric AAV6 particles, as well as the ampicillin resistance gene.

### AAV production and purification and quantification of the genomic titer.

Recombinant AAV6 variants were produced by ViGene Biosciences by triple transfection of adherent growing HEK293 cells with the AAV6 variant RepCap plasmids, helper plasmid, and NanoLuc luciferase donor plasmid using polyethylenimine. The transfected cells were harvested 72 h posttransfection, pelleted, and subjected to three freeze-thaw cycles to release the AAV vectors from the cells. Vectors released into the growth medium during the 72-h incubation period were recovered by addition of polyethylene glycol (PEG) to a final concentration of 8.2% (wt/vol) and subsequent precipitation. The rAAVs from the cell pellet and the PEG precipitate were combined and treated with benzonase for 30 min to 2 h. The raw lysate was clarified by centrifugation and the supernatant purified by iodixanol gradient ultracentrifugation, as previously described (34), using a Beckman VTI 50 rotor at 48,000 rpm for 2 h. The genome-containing capsids were extracted from the 40% iodixanol fraction, which was buffer exchanged and concentrated using an Amicon Ultracel 100-kDa cutoff concentrator column (Millipore).

The packaged genome titers were determined by quantitative PCR (qPCR) using SYBR green stain, with primers directed to the AAV2 inverted terminal repeat regions. The physical particle titer was determined by an AAV6 titration ELISA (PRAAV6; Progen), according to the manufacturer’s instructions, that recognizes a conformational epitope present on AAV6 and all other capsid variants tested here but does not detect unassembled capsid proteins.

### Cell transduction and luciferase assay.

Primary human CD3^+^ and CD8^+^ T cells were isolated from unfractionated peripheral blood mononuclear cells (PBMCs) using the EasySep human T cell isolation kit and human CD8 T cell isolation kit with RapidSpheres (Stemcell Technologies). Mobilized human primary CD34^+^ cells from peripheral blood (obtained from Caltag Medsystems, Buckingham, UK). Both T cells and CD34^+^ HSCs were cultured in X-Vivo 15 medium (Lonza) supplemented with 10% human serum AB (Merck Sigma-Aldrich), 300 IU/ml interleukin 2 (IL-2), and 5 ng/ml IL-7 and IL-15 (PeproTech) at 37°C and 5% CO_2_. Neuro2A and U87 were maintained in Dulbecco’s modified Eagle’s medium (DMEM) supplemented with 10% heat-inactivated fetal calf serum, 50 mM HEPES, 100 U of penicillin/ml, and 100 μg of streptomycin at 37°C in 5% CO_2_. For culturing HMC3 cells, minimal essential medium (MEM) supplemented with 10% heat-inactivated fetal calf serum and 100 U of penicillin/ml and 100 μg of streptomycin was utilized.

For CRISPR plus AAV treatments, 2 × 10^5^ T cells were first stimulated using anti-CD3/CD28 Dynabeads (Invitrogen) in complete T cell medium for 48 h prior to electroporation. T cells were electroporated with 15 μg Cas9 mRNA (TriLink) and 10 μg AAVS1-specific sgRNA using the Neon electroporator (3 × 10^5^ in 10 μl Neon tip) and the following pulse conditions: 1,400 V, 10 ms, and 3 pulses. Electroporated T cells were recovered in T cell medium for 2 h before addition of purified AAV vectors to the media at MOI ranging from 200 to 1 × 10^6^ viral particles. The volume of each virus sample was adjusted by diluting in phosphate-buffered saline (PBS), so each treatment received equivalent volumes, compensating for the lower-concentration vector samples. Medium was replaced after 24 h with fresh complete T cell medium and again every 2 to 3 days. For quantification of T cell transduction, luciferase activity of the transduced T cells was measured at 7, 14, and 21 days post-infection. T cells were harvested, and firefly luciferase was analyzed using the Dual-Glo luciferase assay kit (Promega, Madison, WI) according to the manufacturer’s instructions. Luminescence was measured using a PHERAstar microplate reader (BMG Labtech).

For analysis of the AAV vector transduction efficiency in Neuro2A, U87, and HMC3 cells, 24-well plates were seeded 24 h prior to infection. The cells were infected with purified AAV6, AAV12, variant 5, and variant 5.3 NanoLuc vectors at an MOI of 2 × 10^4^ gc/cell. After 48 h, cells were lysed and luciferase activity assayed using a luciferase assay kit (Promega) as described in the manufacturer’s protocol. Uninfected cells were used as a negative control.

### Investigation of the role of AAP on AAV6 variant production.

For experiments investigating the impact of coexpressing wild-type AAP during viral packaging, AAV production protocols were modified to include polyethylenimine cotransfection of an AAP-6 expression plasmid (ORF under the control of the CMV promoter, synthesized by GenScript Biotech), the variant 5 AAV cap plasmid, and an adenoviral helper plasmid into HEK293 in equivalent amounts, along with the AAVS1-nanoluciferase-targeting vector. Constructs expressing the AAP gene with either the native leucine start codon or a substituted methionine start codon were tested. Viruses produced using expression of AAP in *trans* were purified as described above.

For experiments investigating the reversion back to wild-type AAP-6 within the AAP modified in the variants, additional vectors were designed and synthesized with combinations of amino acid substitutions with residues 13 to 27 of the AAP-6 sequence. Virus preparations packaging the AAVS1-nanoluciferase construct using each AAP-modified variant were generated and evaluated for transduction in human T cells as described above.

### Analysis of capsid protein expression.

For the analysis of VP expression of the different capsid variants, HEK293 cells were transfected as described above, with and without the addition of AAP, and cells were lysed 72 h posttransfection by three cycles of freezing and thawing. The cleared supernatants were denatured for 5 min at 95°C in SDS-containing protein sample buffer and separated on a 10% polyacrylamide-SDS gel. The proteins in the gel were transferred to a nitrocellulose membrane and probed with monoclonal antibody (MAb) B1 (35). The membrane was washed and incubated with an anti-mouse IgG horseradish peroxidase-linked secondary antibody to be detected by enhanced chemiluminescence. For visualization of the incorporation of the individual VPs into the capsids of the AAV variants, the lysates from a total of 10 transfected 15-cm plates of HEK 293 cells were purified by AVB-Sepharose columns as previously described (36) and concentrated using an Apollo 7-ml centrifugation concentrator (Orbital Biosciences, Topsfield, MA). The purified AAV vector preparations were analyzed by SDS-PAGE and the gel stained with GelCode blue stain reagent (Thermo Fisher).
